# Editorial: Translational neuroscience and reverse translational neuroscience: What's giving us hope?

**DOI:** 10.3389/fnins.2023.1149819

**Published:** 2023-02-22

**Authors:** Wael M. Y. Mohamed, Badrah Saeed Alghamdi, Athanasios Alexiou

**Affiliations:** ^1^Basic Medical Science Department, Kulliyyah of Medicine, International Islamic University Malaysia, Kuantan, Pahang, Malaysia; ^2^Department of Physiology, King Abdulaziz University, Jeddah, Saudi Arabia; ^3^Novel Global Community Educational Foundation (NGCEF), Hebersham, NSW, Australia; ^4^AFNP Med, Wien, Austria

**Keywords:** neuroscience, translational, reverse translational research, neurodegeneration, animal model

## Animal models, efficient biomarkers and computational methods in neurodegeneration

Globally, the number of individuals with a neurological condition is growing. Neurological illnesses are the primary cause of disability-adjusted life years and the second largest cause of mortality globally, according to reports. According to the World Health Organization, one in four individuals suffers from a neurological or mental problem, and brain illnesses are thus regarded as one of the biggest hazards to public health.

Neuroscience is one of the most quickly evolving medical and biological disciplines. The previous decade has seen an astonishingly quick accumulation of knowledge on all parts of neuroscience, particularly as they relate to neurological, psychological, and psychiatric sciences. “bench to bedside” defines the standard paradigm of neuroscience research. It's a tried-and-true method consisting of a straight route from fundamental research through clinical trials. However, a more dynamic method may be required for the burgeoning field of neuroscience. Transitioning straight from examining individual cells to testing on people is not possible nor safe. The pivot point is provided by translational research. It bridges the gap between fundamental and clinical research by bringing together a diverse group of experts to develop and accelerate the implementation of a finding. Biomedical research is so intricate, and there is so much accessible information. The communication constantly flows in both directions. It is a continual cycle in which one study finding inspires the next.

The attitude of study that characterized the “Decade of the Brain” throughout the 1990s has propelled tremendous advances in fundamental neurosciences. Slowly but surely, promising uses of this rising understanding are being realized. Previously, biological indicators for mental or cognitive illnesses were deceptive owing to constraints, such as the inability to directly examine the functioning of the human brain. Despite the availability of pharmaceutical medicines and other treatment techniques that successfully manage the symptoms of some of these severe and frequent illnesses, there are few reliable diagnostic indicators. In the booming area of translational neuroscience, there is an extraordinary need to cultivate investigators capable of bridging the fundamental-clinical neuroscience divide, given the disparity between clinical and basic neuroscience.

Neuroscientists have attempted to aid in creating viable methods and applications to assist clinical patients with neurological illnesses; nevertheless, the vast majority of research conducted to date has failed to provide clinically relevant insights. Collaboration between neurologists in clinics, neuropathologists, neuroradiologists, and neuroscientists in wet and dry labs is crucial for highlighting clinical concerns, selecting appropriate methodological approaches, inventing tools and technologies, and translating these findings into patient interventions and treatments. In conjunction with clinicians and neuroscientists in the domains of neurology and neuropsychiatry, the present Research Topic serves to focus the translational neuroscience (bench to bedside) research of the future toward relevant therapeutic results. Although translational research is often envisioned as a progression from fundamental bench research through preclinical model experiments to clinical studies to application, reverse translational research is a methodical investigation of clinical research outcomes in a preclinical model. Simply, it is transitioning from the Bench to the bedside and vice versa.

Trimmel et al. aim to establish an animal model suited for assessing the physiological relationships between cerebrospinal fluid (CSF) dynamics, hemodynamics, and abdominal compartment pressures. To examine interdependent physiologic pressure propagation and multiparameter impacts on CSF dynamics, they developed a unique and comprehensive ovine animal model. The findings of this work will lead to more *in vitro* bench testing, the development of innovative quantitative models, and the creation of a pathological ovine hydrocephalus model.

Similarly, Khandia et al. undertook research to examine the composition, physical characteristics of the protein, and selectional and mutational pressures that influence codon use preferences in a panel of genes directly or indirectly contributing to metabolism and neurodegeneration. Their investigation revealed that selection pressure, compositional restrictions, and mutational factors dominate in determining codon use.

Alqurashi et al. conducted an intriguing investigation to examine the relationship between sleep length and cognitive abilities in older Saudi individuals. Their results indicated that prolonged sleeping was related to a decline in cognitive abilities among senior Saudi citizens. In addition, the majority of their participants did not nap, followed by moderate nappers. This research will shed light on the association between napping practice and cognitive deterioration among older Saudi individuals. The research also sheds light on the need to monitor the behavior of elderly individuals since their prolonged naps may be related to a more severe type of dementia.

In light of this, Alharbi et al. devised research to examine the association between blood vitamin D levels and stroke clinical severity at admission, as well as functional independence and impairment at discharge, in Saudi Arabia. Their findings indicate lower vitamin D levels are related to worsening stroke clinical severity at admission and functional impairment at discharge. Consequently, the blood vitamin D level may predict both the clinical severity of a stroke at admission and its functional impairment after discharge. However, their findings should be taken with care, and further research is necessary to comprehend the underlying pathophysiology and determine if vitamin D supplementation may be employed in preventing or treating stroke.

The narrative review by Alosaimi et al. highlights the primary neurotransmitter dynamics in Parkinson's disease and their function in mediating DBS effects based on preclinical and clinical evidence. Multiple neurotransmitters are implicated in the neuropathology and pathogenesis of Parkinson's disease (PD), including the SNc dopaminergic, LC noradrenergic, pallidal GABAergic, PPN cholinergic, DRN serotoninergic, and STN glutamatergic systems, according to the research. Technical constraints inherent to detecting transmitter release and transmitter-related charges in distant neural locations may explain why neurochemical alterations induced by DBS have not been thoroughly studied in the literature. Optogenetic studies studying the precise effects of neuromodulation on neurotransmitter release are necessary because they would aid in evaluating DBS's cumulative and chronic effects on local and distant neural components. Improving DBS efficacy, providing more accurate targeting, reducing unpleasant effects, and providing more suitable pharmaceutical intervention choices would enhance the overall quality of PD therapy as a result of a more profound knowledge of neurotransmitter dynamics.

## Conclusion

The scientific community has a tremendous need for various contributions to bring together the most prominent researchers, the most recent vital results, and the most significant historical developments in translational neuroscience. The failure of translational research may occur for a variety of different causes. To name just a few examples, there is still a lack of knowledge about the mechanisms behind disease, preclinical models are often inaccurate or unreliable, experiments in animal models often lack proper experimental design and statistical analysis, and clinical studies often use inappropriate endpoints and enroll unrepresentative samples of the population. Recent quantum jumps in the quality and quantity of translational research have been driven by the combination of collaborative efforts and technological advancements. Using today's molecular tools, we can get considerably more information from a patient's tissue sample than was previously possible.

A wide variety of neurological and neuropsychiatric disorders, such as epilepsy, Multiple Sclerosis, Alzheimer's disease, Parkinson's disease, Huntington's disease, amyotrophic lateral sclerosis, addiction, autism spectrum disorder, and others, require immediate solutions. This Topic aims to understand not only the underlying mechanisms of these conditions but also to shed light on the urgent need for clinical field challenges, difficulties, and obstacles that require immediate solutions. To increase translational effectiveness, it is essential to form interdisciplinary teams in which veterinarians play a central role. Furthermore, the importance of comparative studies in lower species, which help with toxicity, target identification, and mechanistic investigations, is often overlooked by researchers and regulators. As a whole, we stress here the need of a paradigm change in patient-oriented neuroscience research and policy.

## Author contributions

All authors listed have made a substantial, direct, and intellectual contribution to the work and approved it for publication.

